# Extramedullary plasmacytoma of the kidney in an HIV-positive patient: A case report

**DOI:** 10.1097/MD.0000000000018422

**Published:** 2019-12-27

**Authors:** Yubing Li, Chundan Wang, Jiashen Yan, Shaobo Jiang

**Affiliations:** a Department of Urology; b Pathology, The First Affiliated Hospital of Zhejiang Chinese Medical University, Zhejiang, Hangzhou, PR China.

**Keywords:** extramedullary plasmacytoma, HIV infection, kidney

## Abstract

**Rationale:**

Extramedullary plasmacytoma (EMP) is a very rare malignant neoplasm arising from clonal proliferation of atypical plasma cells. Most EMPs involve mucosal lymphoid tissue, particularly in the nasopharyngeal area, respiratory tract, and head and neck region. Such neoplasms of the kidney in patients with a human immunodeficiency virus (HIV) infection are extremely rare.

**Patient concerns:**

A 55-year-old male who had been diagnosed with HIV 1 year previously presented with a 2-week history of intermittent right abdominal pain and gross hematuria.

**Diagnoses:**

Ultrasonography and computed tomography detected a mass that occupied the upper half of the right kidney. A clinical diagnosis of a renal tumor was suspected.

**Interventions:**

The patient subsequently underwent a retroperitoneal radical nephrectomy. The postoperative pathological diagnosis was solitary EMP of the kidney. Adjuvant radiation therapy was provided at doses of 50 Gy in 20 fractions.

**Outcomes:**

Currently, the patient is alive and disease free at 7 months postoperatively. To the best of our knowledge, this is the first case of a primary renal EMP in a patient with HIV.

**Lessons:**

The present case illustrates that this rare type of solitary EMP associated with acquired immune deficiency syndrome can occur in the kidney. Additionally, although rare, solitary EMP should be considered in the differential diagnosis of a renal mass in HIV-infected patients.

## 1 Introduction

Extramedullary plasmacytoma (EMP) is a very rare malignant neoplasm arising from clonal proliferation of atypical plasma cells, and is diagnosed when there is a focus of monoclonal plasma cells in the soft tissue in the absence of systemic disease. EMP is characterized as a plasma cell tumor, along with solitary plasmacytoma of the bone and multiple myeloma, which is considered a more advanced stage of the disease.[^1^,^2^] Although EMP frequently affects the head and neck area, any extraosseous organ may be involved^[3]^; however, only a few cases of EMP in the kidney have been reported in the literature.[^4^,^5^,^6^] EMP is extremely rare in HIV-positive patient^[7]^ and it has been found that these patients are younger and they present a greater tendency to develop solitary extramedullary plasmacytoma with atypical clinical evolution and greater aggressiveness of the neoplastic process. To the best of our knowledge, the present study describes the first case of a solitary kidney EMP in a human immunodeficiency virus (HIV)-positive patient.

## 2 Case report

**Figure 1:**
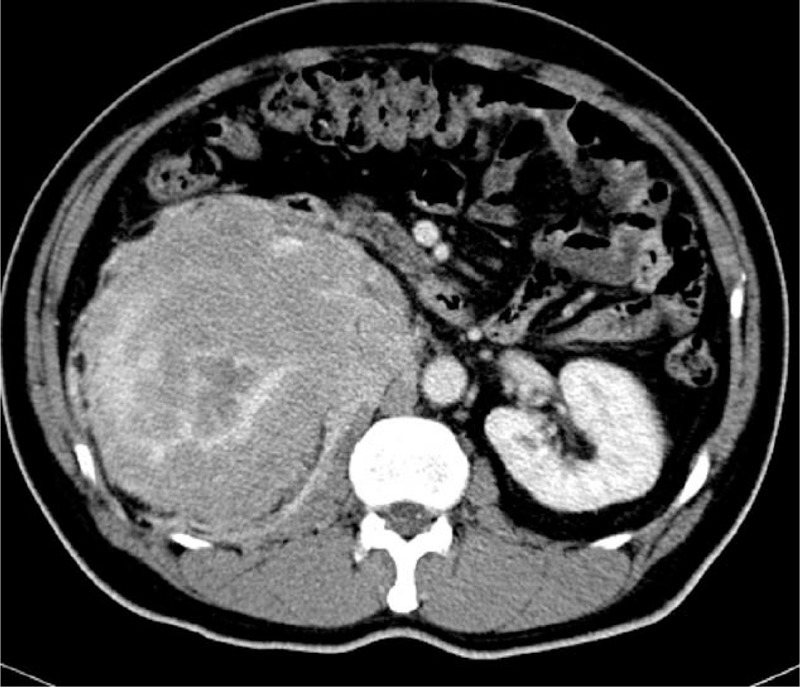
Contrast enhanced computed tomography scan revealing a large heterogeneously enhancing mass involving the right kidney.

**Figure 2:**
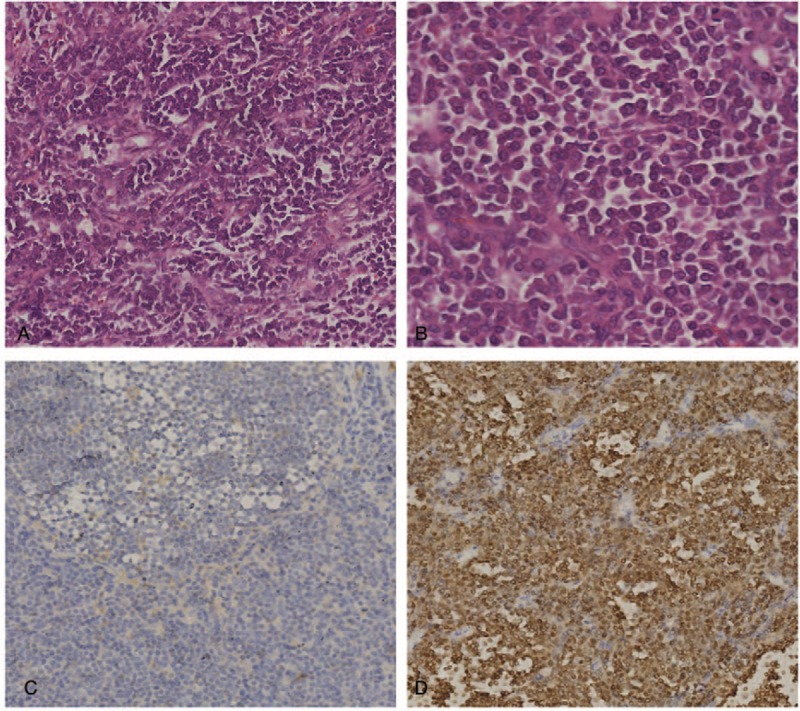
Microscopic examination revealing round monomorphic cells with vesicular and eccentric nucleus and immature plasma cells (A) (hematoxylin and eosin [H&E], ×200). High power view shows plasma cells with basophilic cytoplasm, eccentric nuclei, and typical peripheral condensation of the chromatin (B) (H&E ×400). Immunohistochemical staining shows that the tumor cells were negative for kappa (C) (magnification, ×200) and positive for lambda (D) (magnification, ×400).

A 55-year-old male who had been diagnosed with HIV 1 year previously presented with a 2-week history of intermittent right abdominal pain and gross hematuria. The patient had a 2-year history of sexual intercourse with prostitutes. His medical history included hepatitis B and HIV infections. The HIV infection was well controlled with drugs. He had no significant family, allergic, or smoking history. He had not received any blood transfusions. The physical examination was unremarkable, except for a soft, non-tender mass palpated in his right upper abdomen. A chest X-ray was normal. Ultrasonography detected a mass occupying the upper half of the right kidney. A contrast enhanced computed tomography (CT) scan (Fig. 1) of the abdomen revealed a large heterogeneously enhancing mass measuring ∼14 × 10 cm involving the upper right aspect of the right kidney. A microscopic urinalysis revealed red blood cells, but no white cells, and urine cytology findings were negative for urothelial carcinoma. The complete blood count and electrolyte profile were normal. No abnormalities were detected on a renal function test. No bladder tumor was observed on cystoscopy. We diagnosed the renal tumor as a renal cell carcinoma because of the hematuria, the right renal tumor, and the CT scan findings. A retroperitoneal radical nephrectomy was performed. The surgical margins were clean on histology. A microscopic examination of the tumor section revealed that the tumor was composed of round monomorphic cells with vesicular and eccentric nuclei and immature plasma cells (hematoxylin and eosin [H&E] ×200) (Fig. 2A). A high power view (H&E ×400) revealed plasma cells with basophilic cytoplasm, eccentric nuclei, and typical peripheral condensation of the chromatin (Fig. 2B). Invasion of tumor cells was observed in the pelvic mucosa, renal parenchyma, and perirenal soft tissue of the kidney. Immunohistochemical studies confirmed that the tumor was composed of plasma cells, as evidenced by their reactivity with antibodies to CD138, CD45, vimentin, Lambda light chain, CD79a, and EMA (Fig. 2C). The tumor was negative for Kappa light chain, CD20, CD3, CD56, CD10, smooth muscle actin, and creatine kinase. Further postoperative investigations were performed. No high levels of monoclonal protein were found in the blood or urine. The bone marrow aspiration findings were normal, and a skeletal X-ray revealed no lytic lesions. Eventually, the patient met all of the required clinical and laboratory criteria for a solitary EMP. Adjuvant radiation therapy was given at doses of 50 Gy in 20 fractions. He is alive and disease free 7 months postoperatively.

## 3 Discussion

An EMP is a rare malignant neoplasm usually arising from B-lymphocytes outside the bone marrow. EMP occurs either as a solitary plasmacytoma or as multiple myeloma. The diagnosis of EMP depends upon the demonstration of an extramedullary plasma cell tumor with no evidence of systemic signs and symptoms associated with multiple myeloma.^[2]^ About 80% to 90% of EMP cases involve the mucosa-associated lymphatic tissue of the upper respiratory tract, and 75% of them appear in the nasal and paranasal regions^[8]^; only a few cases of EMP in the kidney have been reported in the literature.[^4^,^5^,^6^] To the best of our knowledge, the present study describes the first case of a solitary kidney EMP in an HIV-positive patient. Such neoplasms in this group of patients are extremely rare.^[9]^ Additionally, this tumor displays different clinical behavior among HIV-positive patients: occurrence in the younger age group has a shorter latency period, often with extramedullary involvement and a more aggressive clinical course with a poor prognosis due to poor immunity of the patient.[^9^,^10^,^11^] Indeed, the mean age of EMP in noninfected patients is 60 years,^[2]^ but in HIV-positive patients the mean age is 33 years.^[11]^ In this study, the patient was 55 years old.

No guidelines are available for solitary EMP associated with HIV due to its rarity and variable presentation, and there are no widely established treatment criteria. Surgery or radiotherapy, alone or in combination, can be used according to tumor size, clinical stage, and willingness of the patient. Plasmacytomas are generally very radiosensitive, and the optimal dose for local control is 40 to 50 Gy (depending on tumor size) delivered over 4 to 6 weeks.^[12]^ Wu et al described the case of an HIV-positive individual with a solitary EMP of the central nervous system that was treated by chemotherapy.^[13]^ Ramadan et al^[14]^ reported a patient with solitary testicular EMP and AIDS who was treated with combined surgical excision and radiotherapy. Cao et al^[9]^ described a case of an HIV-positive individual with solitary adrenal EMP who was treated by retroperitoneal laparoscopy. In the present patient, the renal mass was successfully removed by retroperitoneal radical nephrectomy. Adjuvant radiation therapy was given at doses of 50 Gy in 20 fractions.

Although the relationship between a plasmacytoma and HIV infection remains unclear, multiple myeloma in HIV-infected patients reportedly has an atypical clinical evolution; it tends to present as a solitary bone plasmacytoma or EMP.^[10]^ The pathogenesis of a plasmacytoma in HIV-positive patients is unknown, but chronic antigen stimulation and immunodeficiency are believed to be the driving forces of the process.^[15]^ In view of a high incidence of progression to multiple myeloma in due course, patients should be kept under constant surveillance.^[11]^

## 4 Conclusion

In conclusion, the present study illustrates that this rare type of solitary EMP associated with HIV can occur in the kidney, and that retroperitoneal radical nephrectomy combined with adjuvant radiation therapy may be a good method to manage this tumor. Additionally, although rare, solitary EMP should be considered in the differential diagnosis of a renal mass in HIV-infected patients.

## Acknowledgments

The English in this document has been checked by at least two professional editors, both native speakers of English. For a certificate, please see: http://www.textcheck.com/certificate/fQ5dId

## Author contributions

**Methodology:** Shaobo Jiang.

**Resources:** Chundan Wang.

**Supervision:** Shaobo Jiang.

**Writing – original draft:** Jiashen Yan.

**Writing** – **review & editing:** Yu-bing Li.
